# Impact of the COVID-19 Health Crisis on Key Populations at Higher Risk for, or Living With, HIV or Hepatitis C Virus and People Working With These Populations: Multicountry Community-Based Research Study Protocol (EPIC Program)

**DOI:** 10.2196/45204

**Published:** 2023-12-14

**Authors:** Rosemary M Delabre, Marion Di Ciaccio, Nicolas Lorente, Virginie Villes, Juliana Castro Avila, Adam Yattassaye, César Bonifaz, Amal Ben Moussa, Ingrid-Zaïre Sikitu, Niloufer Khodabocus, Rosa Freitas, Bruno Spire, Maria Amélia Veras, Luis Sagaon-Teyssier, Gabriel Girard, Perrine Roux, Annie Velter, Valérie Delpech, Jade Ghosn, Lucas Riegel, Daniela Rojas Castro

**Affiliations:** 1 Community-based Research Laboratory Coalition PLUS Pantin France; 2 Centre Estudis Epidemiològics sobre les Infeccions de Transmissió Sexual i Sida de Catalunya Agència de Salut Pública de Catalunya Badalona Spain; 3 Centro de Investigación Biomédica en Red de Epidemiología y Salud Pública Madrid Spain; 4 Association pour la Résilience des Communautés pour l’Accès au Développement et à la Santé PLUS Bamako Mali; 5 Corporación Kimirina Quito Ecuador; 6 Association de Lutte Contre le Sida Casablanca Morocco; 7 Association Nationale de Soutien aux Séropositifs et Malades du Sida Bujumbura Burundi; 8 Prévention Information Lutte contre le SIDA Port Louis Mauritius; 9 Grupo de Ativistas em Tratamentos Lisbon Portugal; 10 Aix Marseille Université, Institut national de la santé et de la recherche médicale, Institut de Recherche pour le Développement SESSTIM, Sciences Économiques & Sociales de la Santé & Traitement de l’Information Médicale, Institut des Sciences de la Santé Publique d’Aix-Marseille Université Marseille France; 11 Santa Casa de São Paulo School of Medical Sciences São Paulo Brazil; 12 Santé Publique France Saint-Maurice France; 13 Director of Population and Public Health Directorate North Coast North South Wales Australia; 14 Assistance Publique - Hôpitaux de Paris Nord, Service des Maladies Infectieuses, Center Hospitalier Universitaire Bichât - Claude Bernard Paris France; 15 Infection, Antimicrobials, Modelling, Evolution, Institut National de la Santé et de la Recherche Médicale, Unité Mixte de Recherche 1137, Université Paris Cité Paris France

**Keywords:** COVID-19, key populations, health, crisis, HIV, mobile phone

## Abstract

**Background:**

Information concerning the impact of the COVID-19 health crisis on populations most affected by HIV and hepatitis C virus (HCV; or key populations [KP]), and those working with these populations in community settings, is limited. Community-based organizations working in the field of HIV and viral hepatitis are well placed to identify and meet the new needs of KP owing to the health crisis.

**Objective:**

This study aims to describe the development and implementation of an exploratory and descriptive multicountry, community-based research program, EPIC (*Enquêtes Pour évaluer l’Impact de la crise sanitaire covid en milieu Communautaire*), within an international network of community-based organizations involved in the response to HIV and viral hepatitis. The EPIC program aimed to study the impact of the COVID-19 health crisis on KP or people living with HIV or HCV and people working with these populations at the community level (community health workers [CHWs]) and to identify the key innovations and adaptations in HIV and HCV services.

**Methods:**

A general protocol and study documents were developed and shared within the Coalition PLUS network. The protocol had a built-in flexibility that allowed participating organizations to adapt the study to local needs in terms of the target population and specific themes of interest. Data were collected using surveys or interviews.

**Results:**

From July 2020 to May 2022, a total of 79 organizations participated in the EPIC program. Across 32 countries, 118 studies were conducted: 66 quantitative (n=12,060 among KP or people living with HIV or people living with HCV and n=811 among CHWs) and 52 qualitative (n=766 among KP or people living with HIV or people living with HCV and n=136 among CHWs).

**Conclusions:**

The results of the EPIC program will provide data to describe the impact of the health crisis on KP and CHWs and identify their emerging needs. Documentation of innovative solutions that were put into place in this context may help improve the provision of services after COVID-19 and for future health crises.

**International Registered Report Identifier (IRRID):**

DERR1-10.2196/45204

## Introduction

### Context

Numerous COVID-19 research and epidemiological surveillance data have shown that certain populations are disproportionately affected by the COVID-19 health crisis not only in terms of mortality and morbidity but also in terms of the social and economic consequences of the crisis [1-4]. This is notably the case among the homeless and people living in inadequate or poor housing conditions, which makes the implementation of prevention measures such as social distancing and frequent hand washing difficult or unfeasible [5]. In addition, key populations (KP; such as men who have sex with men [MSM], people who inject drugs, sex workers [SWs], transgender women, and migrants) who are exposed or living with HIV or hepatitis C virus (HCV) may also experience compounding effects of the health crisis in terms of infringement on their human rights and exacerbation of preexisting health inequalities.

International organizations and community-based associations have alerted public authorities to the susceptibility of these marginalized groups in the early phase of the COVID-19 pandemic and the consequences on the HIV response [6]. The HIV epidemic is largely concentrated within KP: MSM, people who inject drugs, SWs and their clients, and transgender women [7]. The lack of consideration or consultation with KP has resulted in further social exclusion [3] and greater susceptibility to violence and human rights abuses [6]. For example, governmental measures such as lockdowns have exposed SWs to great economic instability, forcing them to put their health in danger [8]. The crisis may also have an important impact among transgender populations in terms of reinforcing preexisting inequalities with regard to access to health care, including gender affirming care and social and mental support [9]. Disruptions in access to drug services and clean drug consumption-related equipment were experienced early in the pandemic, possibly increasing the risk of infection with SARS-CoV-2 in addition to HIV and hepatitis B and C among people who use drugs [10-12]. Increased alcohol and drug consumption because of distress may also be a consequence of the crisis, and whether this persists after COVID-19 is of concern [5,13]. Among MSM, and likely other KP, a reduction in sexual activity was observed early in the pandemic owing to lockdowns and distancing measures, which may have resulted in a lower uptake of prevention tools such as preexposure prophylaxis and HIV testing [14,15]. It is thus important to ensure the necessary support and access to services when sexual activity resumes. Finally, disruptions in treatment and other services for people living with HIV and people living with HCV could lead to significant increases in new infections and mortality in the coming years, hindering global elimination efforts [16-18].

The COVID-19 crisis thereby represents a threat to the global progress against HIV and viral hepatitis and has consequently highlighted the importance of engaging or re-engaging and maintaining KPs, people living with HIV and people living with HCV in the HIV and HCV prevention and care pathway. The crisis has in many ways served as a catalyst for the development of innovative solutions for health service delivery since the beginning of the COVID-19 pandemic. For example, many in-person services moved to the web [19], and although this may be a viable solution for some individuals, there remains a concern that remote delivery of care will further exacerbate social gradients of health owing to limited or no access to internet, technological resources, and technological literacy [20]. Efforts to maintain harm reduction services despite lockdowns and other restrictions created an opportunity in some countries to develop or expand multimonth dispensing of opioid agonist treatment, unsupervised dosing and diversification of points of dispensation (pharmacy, vending machines), and take-home naloxone, where they were previously not permitted [21,22]. Similarly, home delivery, multimonth dispensing, and “fast track” antiretroviral therapy refills were solutions put into place in some countries to maintain antiretroviral therapy services [23].

Many of these innovative solutions were put into place by community-based organizations (CBOs), who were notably at the forefront in the early days of the pandemic [21,24-26]. However, studies of the other epidemics have shown a negative impact (depression and stigmatization) of health crises on health workers [27,28]. Given their proximity to KP, and their essential role in the provision of support, prevention, and treatment for these populations, it is equally important to study the impact of the COVID-19 health crisis on community health workers (CHWs) [29].

The COVID-19 pandemic has thus reinforced the necessity of community engagement to provide adapted responses to the specific needs of KP. Community organizations worldwide have effectively led initiatives to provide COVID-19 information and share expertise [24]; however, data regarding these initiatives are limited. Therefore, it is important to document the new solutions put in place during the COVID-19 health crisis and to document the perceptions and experiences of CHWs.

### Objectives

The EPIC (*Enquêtes Pour évaluer l’Impact de la crise sanitaire covid en milieu Communautaire*) community-based research program aimed to understand how the COVID-19 health crisis affected the populations at higher risk for, or living with, HIV and viral hepatitis and the people working with these populations at the community level, and to identify the key innovations and adaptations in HIV- and hepatitis-related services. In the following sections, we describe the general framework of the EPIC program, which was implemented in 32 countries across the world in partnership with local CBOs involved in the fight against HIV and viral hepatitis. The program was developed by Coalition PLUS, an international union of CBOs involved in the fight against HIV and viral hepatitis, in partnership with external scientific partners. Founded in 2008, Coalition PLUS unites 16 member organizations and >100 partner organizations in 52 countries. These organizations are grouped into 6 regional networks (Middle East and North Africa [MENA], West Africa, Central and East Africa, Indian Ocean, Americas-Caribbean, and Europe), 2 thematic networks (RIGHT PLUS [research] and AGCS PLUS [advocacy]), and a linguistic (Lusophone) network.

## Methods

### General Design and Study Development

**Figure 1:**
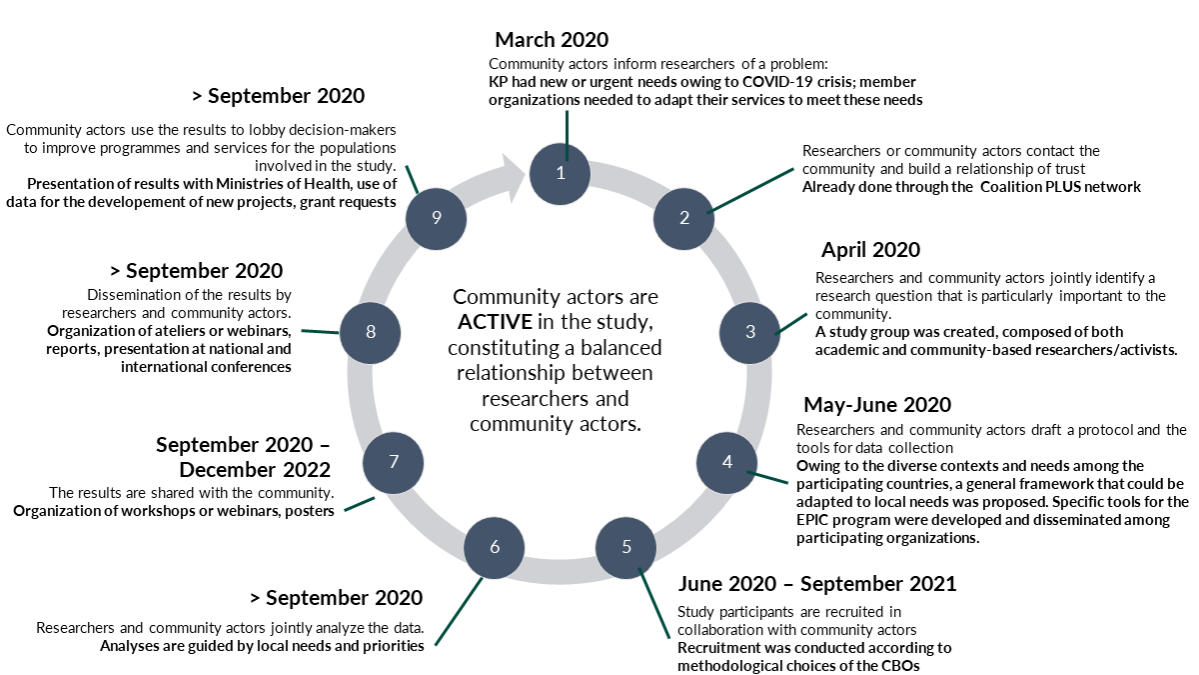
Stages of a community-based research project adapted to the EPIC (*Enquêtes Pour évaluer l’Impact de la crise sanitaire covid en milieu Communautaire*) program. CBO: community-based organization; KP: key populations.

EPIC is an exploratory and descriptive multicountry, cross-sectional, and community-based program that uses mixed methods: qualitative and quantitative. To accommodate the diverse themes of interest and target populations, as well as the exceptional circumstances, the study was customizable at the local level, and there was a common basis for all organizations implementing EPIC. However, data collection tools were adjustable depending on the target population or populations and specific themes of interest, which were determined by the implementing organizations. Other considerations such as human and financial resources and technical capacity of the implementing organizations were also taken into consideration. The adaptative methods of the EPIC program thus resulted from an urgent need to document the COVID-19 crisis within the Coalition PLUS network and adapt to the realities of the field within the context of an ongoing pandemic. This decision was in line with the community-based approach and with Allport [30] when he postulated that “we should adapt our methods so far as we can to the object, and not define the object in terms of our faulty methods.” Following the community-based approach [31], many CBOs, members or partners of Coalition PLUS, actively participated at all stages of this research project (Figure 1).

Starting in March 2020, Coalition PLUS members and partners mobilized to respond to the COVID-19 crisis within their organizations and countries. These CBOs were confronted with new needs such as supplying CHWs and beneficiaries with protective equipment and information regarding prevention measures, adapting services to respect governmental measures (lockdowns, distancing, and mobility restrictions), and provision of basic needs such as food for beneficiaries. In this context, there was a growing concern that KP at higher risk for HIV or HCV, and people living with HIV or HCV, were likely to have the greatest difficulty adapting to public health measures and most likely to experience negative impacts of the COVID-19 crisis.

On April 23, 2020, the Community-based Research Laboratory (CBRL) at Coalition PLUS organized a web-based Zoom (Zoom Video Communications, Inc) meeting with representatives from each of the regional, thematic, and linguistic networks as well as external scientific partners (hereafter, the EPIC study working group). A total of 18 people participated in this meeting and an additional meeting was held on April 28, 2020, with 5 people who were unable to join the first meeting. The aim of these meetings was to set the objectives of the project and the methodology that was best adapted to the current context and timeline. To better identify the needs within the various Coalition PLUS networks, the working group then consulted with the member and partner organizations of their respective networks to identify common themes. The feedback of these consultations informed the draft proposal of the research project, which was shared at a web-based Zoom meeting held on May 13, 2020. A total of 24 people participated in this meeting. Following this meeting, and individual exchanges with the working group members, the final study documents were finalized and shared on June 12, 2020.

### Objectives

The EPIC program had 3 main objectives:

1. To study the impact of the COVID-19 health crisis on KP at higher risk for, or living with, HIV or HCV (people who use or inject drugs, SWs, MSM, transgender people, and migrants)
2. To study the impact of the COVID-19 health crisis on people working with these populations at the community level (peer educators, CHWs)
3. To identify the key innovations and adaptations in HIV and HCV services that could be maintained and extended beyond the COVID-19 health crisis.

### Study Population and Inclusion Criteria

The inclusion and exclusion criteria of the EPIC program are presented in Textbox 1.

**EPIC (*Enquêtes Pour évaluer l’Impact de la crise sanitaire covid en milieu Communautaire*) program inclusion and exclusion criteria.**

**Inclusion criteria**

- Part of at least one of the eligible study populations: people living with HIV or hepatitis C virus, people who use or inject drugs, sex workers, men who have sex with men, transgender people, migrants, community health workers
- Above the age of the majority in the recruitment country
- Accept to participate and provide informed consent

**Exclusion criteria**

- Not a part of one of the eligible study populations
- Below the age of the majority in the recruitment country
- Refuse to participate or provide informed consent

### Nomenclature

As the implementation of the EPIC protocol varied among the participating countries, based on target population, themes of interest, and methodology used, the overall project is referred to as the EPIC program. EPIC study or studies refer to the local implementation of the program among a specific target population. Therefore, in countries with multiple target populations, the studies were referred to as EPIC-(Methodology) (Country name) (Target Population).

### Data Collection Tools

#### Quantitative Data

Two structured questionnaires were designed to collect quantitative data: one questionnaire targeted KP and the other targeted CHWs. Both questionnaires were organized into different modules so that each organization had the option to implement modules corresponding to their specific interests and target populations. Textbox 2 describes the modules of each questionnaire. There was one mandatory module in the KP questionnaire and 3 mandatory modules in the CHWs questionnaire. Additional questions could be added by each participating organization to collect information on a specific topic that was not already covered by the questionnaire or questionnaires. It was not possible to change the wording of the questions or the proposed responses.

To centralize all the quantitative EPIC data, both questionnaires were created in the Voxco survey software, offering not only web-based but also offline data collection (via a tablet or smartphone app). Organizations that collected data using paper questionnaires entered the data into the Voxco software.

**Modules of the key populations and community health workers questionnaires.**

**Key populations**

- Sociodemographic, general impact of COVID-19 and intention to vaccinate against COVID-19 (mandatory module)
- Sexual activity and prevention strategies for HIV and other sexually transmitted infections
- Experience and application of lockdown or barrier measures
- Socioeconomic needs and responses in relation to the COVID-19 health crisis
- Representations and perception of the COVID-19 risk
- Well-being and resilience
- Access to health care
- COVID-19 impact on pre-exposure prophylaxis
- People who use or inject drugs
- People living with hepatitis C virus
- People living with HIV
- Sex workers
- Migrant

**Community health workers**

- Sociodemographic, general impact of COVID-19 and intention to vaccinate against COVID-19 (mandatory module)
- Experience of the COVID-19 crisis at work (mandatory module)
- Experience and application of lockdown or barrier measures
- Relationship with beneficiaries (mandatory module)
- Representation and perception of COVID-19 risk
- Well-being and resilience

#### Qualitative Data

Two semistructured interview guides were designed to conduct individual interviews with the KP of member and partner organizations and with the CHWs of these organizations. An additional interview guide was designed for CHWs who also belonged to or identified with a KP group. Individual interviews aimed to explore in greater detail the experiences and needs of KP communities during the COVID-19 crisis. Textbox 3 describes the main areas of interest covered by the interview guides.

Interviews were conducted in different ways to accommodate the local restrictions in effect during the data collection period: face to face, on the web (with or without video), or by phone.

All interviews were conducted by trained CHWs, members of the Coalition PLUS community-based research team, or by a local consultant. The interview guides were translated into local language or languages in collaboration with local partners. Interviews were conducted in the local language or languages and recorded using a voice recorder. Voice recordings were transcribed verbatim and translated into French or Spanish, if necessary, for analysis. To guarantee the confidentiality and the anonymity of the interview, external consultants conducted all the interviews among CHWs.

**Main topics covered by the interview guides for key populations, community health workers, and community health workers who identify with a key population.**

**Key populations**

- COVID-19 knowledge, risk perception, and experience of the COVID-19 crisis (in terms of stigma, emerging needs)
- Attitudes toward COVID-19 vaccination
- Experience of lockdown and other governmental measures
- Overall health care access
- Relations with the Coalition PLUS member or partner structure

**Community health workers**

- Personal experience of the COVID-19 crisis within the professional sphere
- Attitudes toward COVID-19 vaccination
- Impact of the COVID-19 health crisis on the beneficiaries

**Community health workers who identify with a key population**

- Personal experience of the COVID-19 crisis within the professional sphere
- Attitudes toward COVID-19 vaccination
- Impact of the COVID-19 health crisis on the beneficiaries
- Experience of lockdown and other governmental measures

#### Complementary Data

A country information sheet concerning local policies, government measures taken during the health crisis, and COVID-19–related data (eg, incidence at study launch) was created to gather information about the context in which the quantitative or qualitative data were collected. Similarly, an organizational information sheet was created to collect general information about organizations that implemented EPIC among CHWs (eg, size of the organization, KP reached, and regular and COVID-19–related activities).

### Sample Size

Given the COVID-19 context and the exploratory and descriptive aims of the project, convenience samples were more adapted to the uncertain pandemic context than samples based on statistical criteria. Indeed, the aim was to gather information about the impact of the COVID-19 crisis on KP and CHW in many Coalition PLUS members and partner organizations. Each organization has its own specificities regarding the COVID-19 crisis at the local level, number of beneficiaries or users reached, and number of CHWs involved. However, before the implementation of the EPIC study, each organization provided an estimation of the sample size to be recruited on the basis of their context and their experience with the target populations to be included in the qualitative or qualitative studies. This sample size estimation was important to orientate or validate the methodological choices through technical support from the Coalition PLUS research team. As a general guide, a minimum of 100 respondents was recommended for quantitative studies and 10 for qualitative studies. Organizations that targeted <100 persons for quantitative studies were encouraged to switch to a qualitative methodology.

### Ethical Considerations

Regarding data protection regulations, the EPIC protocol was registered at the French National Commission of Informatics and Liberties (registration #2218347 v 0).

Local ethics approval was obtained in each participating country before study implementation (Multimedia Appendix 1).

Before taking part in the study, participants were provided information regarding the research objectives, how the data were collected, managed, and by whom, as well as the participant’s rights. For web-based participation, this information was available on the landing page of the EPIC questionnaire in Voxco. Participants had access to the questionnaire once they provided their consent to participate. For face-to-face participation, oral consent was obtained after this information was delivered and before starting the questionnaire or the interview. Face-to-face interviews were conducted in places that guaranteed anonymity and privacy.

All participating organizations signed a cooperation agreement to guarantee the security of the data collected in compliance with both French data protection regulations and local ethics and data protection requirements. The cooperation agreement also detailed collaborative research guidelines and operating rules for teams involved in the study, such as guaranteeing the place of communities in the research process, use of Voxco software, and the EPIC program logo.

### General Coordination of the EPIC Program

The overall coordination of the EPIC program was assured by the Coalition PLUS CBRL, which initially comprised 7 people in 2020, including the principal investigator of the EPIC program and the Director of the Laboratory, 1 research manager, 4 research officers, and 1 biostatistician. By 2021, the team expanded to include an additional research manager, 6 research officers, and 1 data manager for a total of 15 team members. One research officer was dedicated full-time to the coordination of the EPIC program, whereas the other team members worked on the EPIC program in addition to other studies. The follow-up of all participating organizations, in terms of presentation of the study protocol and other documents, development of local adaptation of the study tools, and overall technical support throughout the entire research process was provided by the CBRL team. In 2020, each participating organization was followed through regular Zoom calls and email exchanges by 2 members of the initial team. The addition of the new members in 2021 allowed a more local follow-up of participating organizations as 5 research officers were based in 5 Coalition PLUS regional networks (MENA, West Africa, Central and East Africa, Indian Ocean, and Americas-Caribbean).

### Local Implementation and Data Collection

Following the community-based approach, and within the framework of the capacity-building mission of Coalition PLUS, each member or partner organization willing to locally implement EPIC was assisted by at least one person of the CBRL to guide, check, and validate all steps of the study implementation. Regarding quantitative data collection, each participating member or partner organization was trained on the Voxco software from the CBRL to learn the basic functionalities of the software, for example, how to modify a questionnaire, make it available on the web, and follow-up recruitment. All questionnaires were checked and validated by a member of the CBRL before the survey was launched. Regarding qualitative data collection, all CHWs who conducted interviews received training through a video conference from a qualitative researcher from the CBRL. Each local organization that used qualitative methods sent the first audio interviews and transcriptions to CBRL to check the quality of the interviews. If needed, additional support was provided by the CBRL to improve the quality.

It was crucial that the implementation of the research study did not become a barrier to the continuity of member and partner organizations’ services during the health crisis. Therefore, implementation had to match the organization’s needs in terms of data collection and topics of main interest while considering their local constraints (available human resources, contact with KP, COVID-19 prevention measures, etc). The local design and implementation of the EPIC protocol was documented in the EPIC implementation document. Textbox 4 describes the main sections of the implementation document and its content. The completed implementation document was used as a basis for the local EPIC protocol and was submitted to the local ethics committee in addition to the general protocol.

Once the implementation document was completed, the participating organizations translated all the EPIC study tools into the local language when necessary. The CBRL provided the EPIC main study documents (questionnaires, including web-based questionnaire in Voxco, interview guide, informed consent, etc) in 4 languages: English, French, Portuguese, and Spanish. The study documents were shared with external scientific partners upon request.

Each participating organization was responsible for the survey promotion and the entire data collection process, including data storage when not using the Voxco software (pen-and-paper questionnaires, voice recordings, etc).

**Content of the EPIC (*Enquêtes Pour évaluer l’Impact de la crise sanitaire covid en milieu Communautaire*) implementation document.**

**Section and main information of the section**

1. Collaborators

- Local reference person responsible for the local implementation of the EPIC study
- Name and roles of all involved collaborators
- Budget (if any)

2. Local context

- General introduction and rationale of the project: COVID-19 situation, affected population, etc
- Brief presentation of the organization: history, key populations, etc
- Reasons for implementing EPIC, target populations, etc

3. Objectives

- Selection of the objectives to be used by the organization

4. Study population: List of all key populations to be surveyed by the organization, among

- People who use or inject drugs
- Sex workers
- Men who have sex with men
- Transgender people
- People living with HIV
- People living with hepatitis C virus
- Migrant people
- Community health workers

5. Inclusion criteria

- Be a part of the study population, above the national age of majority, and accept to participate and provide informed consent

6. Ethics committees

- French National Commission of Informatics and Liberties and local ethics committee approvals

7. Survey methods

- Quantitative surveys:
  - Study population or populations
  - Modules of the questionnaire to be used and additional questions (if any)
  - Administration of the questionnaire: face-to-face (pen-and-paper and tablet offline), self-administered (pen-and-paper, tablet offline, and web-based questionnaire)
  - Recruitment strategies: promotion (specific venues and on the web), key persons to be contacted and involved, places of recruitment, other channels for promotion or recruitment
  - Estimated time of recruitment and expected sample sizes
- Qualitative surveys
  - Target population or populations
  - Expected sample sizes
  - Recruitment strategies
  - Interview characteristics: who will conduct the interviews, where, face-to-face or remotely, etc

8. Informed consent

- Brief note presenting the survey and participants’ legal rights (voluntary participation, right to drop out at any moment, anonymity and privacy, and data storage)
- Ways to obtain consent: oral, voice recorded, or tick box

9. Data management

- Places where the data will be stored
- People granted with access to these data

### Data Management

#### Overview

All data (quantitative, qualitative, and complementary) were centralized at Coalition PLUS and were securely stored and transferred. No personal data were collected to ensure anonymity of the study participants. The European General Data Protection Regulation requirements in terms of data protection are complied with regard to data processing and storage.

#### Quantitative Data

Quantitative data that were not collected through Voxco (ie, pen-and-paper questionnaires) were entered into Voxco by the corresponding organization, and hard copies of the questionnaires were stored in a secure place with restricted access. All databases generated by Voxco were then cleaned by the CBRL and shared with each participating organization. All databases are stored on a secured server with access restricted to the CBRL biostatistician and data manager. Each participating organization had access to its own data. The global or subset of the global database may be accessed if a formal demand to perform multicountry analyses is approved by the EPIC study group team.

#### Qualitative Data

Voice recordings, verbatim transcripts, and translations (when necessary) were sent by a secure link to the CBRL and stored.

### Analyses

#### Overview

In general, local level analyses are prioritized as the overall aim of this study was to collect information regarding the impact of the COVID-19 crisis on KP and to identify specific needs to inform local or national interventions or adaptation of services. Analyses at the global and regional levels, and within specific populations, will also be conducted.

#### Quantitative Analyses

All participating organizations were provided with a cleaned database and descriptive tables of all their data, which were prepared by the CBRL. Technical assistance may also be proposed for conducting more in-depth analyses. Participating organizations are also encouraged to seek partnerships with local universities if support is needed for the analyses.

Country-level analyses will be conducted so that participating organizations can build on national results. When possible, multicountry analyses will be conducted, grouping countries by language, geographical region, key issues (eg, health care access), or targeted KP.

Descriptive analyses will be conducted to first characterize the needs of the participants. Three validated scales were used in this study: Patient Health Questionnaire-9 for depression [32] and the Generalized Anxiety Disorder-7 for anxiety [33] and the 6-item Brief Resilience Scale (BRS) for resilience.

#### Qualitative Analyses

According to the quality of qualitative data collected, 2 analysis methods are planned:

1. For corpus with a good quality of data collection, a thematic content analysis will be conducted on the transcripts of the interviews by the local consultant or consultants or CBRL in close collaboration with the local partners. The objective of the analysis will be to identify most relevant themes to describe the impact and experiences of the COVID-19 crisis in collaboration with the partners. The validity of the analysis will be checked with the interviewers or local consultant or consultants.
2. For corpus with difficulties in qualitative data collection (concerning several local organizations for which this was their first time using qualitative methods), a descriptive of the results will be provided by the CBRL based on quotes and summary themes. In addition, this experience will be used to strengthen partners’ abilities in qualitative methods through working sessions to discuss the lessons learned and identify points of improvement.

The quality of the interviews was evaluated by the CBRL based on the transcriptions received. The criteria used were (1) the respect of the interview guide, (2) the quality of the exchanges (interviewers follow the semistructured method and had nonjudgmental and nondirective attitude during the interview), and (3) the quality of the transcription (all the interactions were transcribed).

#### Publications and Scientific Dissemination

All participating organizations are strongly encouraged to disseminate the results of the EPIC studies through various formats and channels of communication that are best adapted to the local context and target audience (including but not limited to the concerned communities and other stakeholders). EPIC data will be disseminated through webinars, in-person workshops, national reports (eg, detailed study reports and infographics), and scientific publications (article and conference presentations). Knowledge management documents that explore the EPIC program as an example of the community-based research approach, and its implementation, during this health crisis are also under development.

## Results

### General Results

The first EPIC surveys were deployed by the CBOs AIDES (France), the Association de lutte contre le Sida (ALCS; Morocco), Asociația Română Anti-SIDA (ARAS; Romania), and Grupo de Ativistas em Tratamentos (GAT; Portugal) in the summer of 2020. To date, the EPIC program has been implemented by 79 organizations in 32 countries worldwide. In total, 118 country-level surveys were conducted, including 66 quantitative studies and 52 qualitative studies. The details on the number of participants, studies, and countries per target population are described in Tables 1-3.

In addition to Coalition PLUS member and partner organizations, external scientific partners (n=2) integrated specific questions or entire modules from the EPIC questionnaires into dedicated or follow-up questionnaires within the framework of ongoing studies. The data from one of these studies may or may not be included in the global database (and the global analyses) depending on the ability to fusion this specific database with the global database and confirmation that there is no risk of duplication. The study, which was implemented in Togo, Burkina Faso, Mali, and the Ivory Coast, has therefore been excluded from the numbers presented here (Tables 1 and 3).

Quantitative data collection completed on March 15, 2022, and qualitative studies completed on May 31, 2022. A total of 13,773 participants participated in the EPIC program (n=12,871, 93.5% participants for the quantitative studies and n=902, 6.5% participants for the qualitative studies).

To date, all participating organizations have received their cleaned database and descriptive tables. Although some countries have already started to disseminate initial results and work on more advanced analyses, many are starting to conduct or discuss the preliminary analyses. Regarding the diffusion of the results, at least 27 organizations have created infographics with a selection of descriptive data from their EPIC studies, which have also served as a basis for feedback to the concerned communities during dedicated meetings. Organizations are also working on national reports or other documents that will summarize the collected data at the national level. The EPIC data have also been used as an evidence base for discussions with local ministries of health or other governmental institutions.

Description of the EPIC program structure and preliminary global results have been presented at regional or international conferences (n=7). Reflections on the development of the EPIC program as a community-based research study in the context of a pandemic have been published [34].

**Table 1:** Distribution of collected quantitative data by targeted study population (N=12,871 respondents; 66 studies in 29 countries)^a^.

Targeted study population^b^	Respondents (n=12,871), n (%)	Different studies (n=66), n (%)	Countries implicated, n	Countries (n=29)
Community health workers	811 (6.3)	10 (15.2)	9	Benin, Burkina Faso, Burundi, Colombia, Guatemala, Malaysia, Mali, Senegal, Spain
People living with HIV	3923 (30.5)	15 (22.7)	15	Brazil, Burkina Faso, Burundi, Canada (Quebec), Cape Verde, Chile, Guinea-Bissau, Mali, Mauritania, Mauritius, Mozambique, Portugal, Romania, São Tomé and Príncipe, Senegal
Men who have sex with men	2965 (23)	11 (16.7)	10	Algeria, Benin, Burkina Faso, Guatemala, Lebanon, Mali, Peru, Portugal, Senegal, Tunisia
Sex workers	2589 (20.1)	15^c^ (22.7)	15^c^	Argentina, Algeria, Angola, Brazil, Burkina Faso, Cape Verde, France, Guinea-Bissau, Mali, Morocco, Mozambique, Portugal, São Tomé and Príncipe, Senegal, Spain
People who use drugs	1364 (10.6)	10 (15.2)	9	Algeria, Burkina Faso, Cape Verde, Colombia, France, Malaysia, Mozambique, Portugal, Senegal
Migrants	554 (4.3)	2 (3)	2	Colombia, Portugal
Transgender people	324 (2.5)	2 (3)	2	Benin, Timor
Key populations (including sex workers, people living with HIV, men who have sex with men, migrants)^d^	341 (2.7)	1 (1.5)	1	Colombia

^a^Excludes participants from 4 countries (Togo, Burkina Faso, Mali, and Ivory Coast) who participated in the EPIC program within the framework of an ongoing cohort study (CohMSM PrEP). The integration of the data from this study with a global database must be determined. Associations in Mali and Burkina Faso also implemented EPIC studies outside the cohort study and were therefore included in this study.

^b^None of the participating organizations specifically targeted people living with hepatitis C virus for their studies.

^c^Includes study (HIVITS-TS-COVID) conducted in Spain.

^d^No specified target population within the key populations.

**Table 2:** Distribution of collected qualitative data by targeted study population (N=902 respondents; 52 studies in 25 countries).

Targeted study population^a^	Respondents (n=902), n (%)	Different studies (n=52), n (%)	Countries implicated, n	Countries (n=25)
Community health workers	136 (15.1)	9 (17.3)	9	Burkina Faso, Benin, Brazil, Burundi, Guatemala, Lebanon, Malaysia, Mauritania, Senegal
People living with HIV	154 (17.1)	9 (17.3)	9	Algeria, Angola, Cape Verde, Guinea-Bissau, Morocco, Portugal, Tunisia, São Tomé and Príncipe, Mozambique
Men who have sex with men	120 (13.3)	8 (15.4)	8	Angola, Benin, Guatemala, Lebanon, Morocco, Peru, Portugal, São Tomé and Príncipe
Sex workers	186 (20.6)	10 (19.2)	10	Bolivia, Brazil, Canada (Quebec), Cape Verde, Guinea-Bissau, Mozambique, Portugal, São Tomé and Príncipe, Spain, Tunisia
People who use drugs	114 (12.6)	8 (15.4)	8	Algeria, Angola, Canada (Quebec), Cape Verde, Guinea-Bissau, Malaysia, Mozambique, Portugal
Migrants	64 (7.1)	2 (3.8)	2	Colombia, Portugal
Transgender people	39 (4.3)	3 (5.8)	3	Benin, Peru, Timor
Key populations (including sex workers, people living with HIV, men who have sex with men, people who inject drugs, migrants)^b^	89 (9.9)	3 (5.8)	3	Burkina Faso, France, Senegal

^a^None of the participating organizations specifically targeted people living with hepatitis C virus for their studies.

^b^No specified target population within the key populations.

**Table 3:** Distribution of collected quantitative and qualitative data by country^a^.

Countries	Quantitative data (N=12,871), n (%)	Qualitative data (N=902), n (%)
Algeria	481 (3.74)	57 (6.32)
Angola	101 (0.78)	50 (5.54)
Argentina	256 (1.99)	N/A^b^
Benin	888 (6.9)	48 (5.32)
Bolivia	N/A	29 (3.22)
Brazil	246 (1.91)	60 (6.65)
Burkina Faso	838 (6.51)	60 (6.65)
Burundi	432 (3.36)	30 (3.32)
Canada (Quebec)	182 (1.41)	19 (2.11)
Cape Verde	292 (2.27)	30 (3.32)
Chile	300 (2.33)	N/A
Colombia	842 (6.54)	54 (6)
France	347 (2.69)	10 (1.11)
Guatemala	436 (3.39)	49 (5.43)
Guinea-Bissau	533 (4.14)	40 (4.43)
Lebanon	118 (0.92)	30 (3.32)
Malaysia	305 (2.37)	30 (3.32)
Mali	1168 (9.07)	N/A
Mauritania	151 (1.17)	10 (1.11)
Mauritius	260 (2.02)	N/A
Morocco	119 (0.92)	40 (4.43)
Mozambique	705 (5.48)	30 (3.32)
Peru	476 (3.7)	19 (2.11)
Portugal	1381 (10.73)	49 (5.43)
Romania	103 (0.8)	N/A
São Tomé and Príncipe	599 (4.65)	40 (4.43)
Senegal	623 (4.84)	45 (5)
Spain	437 (3.4)	29 (3.22)
Timor	100 (0.78)	10 (1.11)
Tunisia	152 (1.18)	34 (3.77)

^a^Excludes participants from 4 countries (Togo, Burkina Faso, Mali, and Ivory Coast) who participated in the EPIC program within the framework of an ongoing cohort study (CohMSM PrEP). The integration of the data from this study with a global database must be determined. Associations in Mali and Burkina Faso also implemented EPIC studies outside the cohort study and were therefore included in this study.

^b^N/A: not applicable.

### Capacity Building Through the EPIC Program

A total of 139 people were trained during 38 training sessions, amounting to 54 hours of training. Specifically, 29 sessions concerned training on the Voxco platform and the use of its offline functionality, and 2 quantitative data analysis trainings were conducted. Three qualitative training sessions were conducted among 7 associations with 11 people.

Furthermore, several webinars have been organized to encourage sharing of experiences and mutual capacity building between the organizations implementing EPIC such as a webinar among all Spanish-speaking organizations to share experiences and tips regarding practical implementation and recruitment and another webinar for participating organizations in the MENA region, which focused on qualitative methodology and data collection. During the 11th Francophone Conference on HIV/AIDS, AFRAVIH, which was held in Marseille from April 6 to 9, 2022, a round table was organized for participating organizations and academic and community-based researchers to discuss the EPIC program, its implications, and the lessons learned.

## Discussion

### Principal Findings

In response to the COVID-19 health crisis, a general community-based research protocol was rapidly developed and made available to CBOs within the Coalition PLUS network. The research protocol was also adapted by external scientific partners to document the impact of the crisis among their ongoing studies. The aims of the global EPIC program were to identify and respond to the specific needs of KP at higher risk for, or living with, HIV or HCV, as well as those who work with these populations.

With a total of 66 surveys (12,871 respondents) and 52 qualitative studies (902 respondents), the EPIC program may largely be considered a success because of the mobilization of an unprecedented number of member and partner organizations across different networks within Coalition PLUS. The EPIC research program will contribute to wider reflections on developing and implementing research projects that respect the community-based research principles within a pandemic context [35]. Furthermore, the program has collected a consequential amount of data among populations who are not represented in general population survey data, who are often not adequately reached by traditional health structures, and who have specific needs [1,25,36].

Although the analysis of the study data is ongoing, we can reflect on some key elements and lessons learned from the implementation of the EPIC program.

### Community-Based Research

Following the community-based research approach [31], the EPIC program was initiated based on information from CBOs of the emerging and urgent needs of KP at the start of the COVID-19 health crisis. CBOs were, and continue to be, involved at every step of the research study process as detailed in Figure 1.

Although the development and implementation of the EPIC program followed the stages of a community-based research project, this process was accelerated owing to the urgency of the COVID-19 health crisis (7 weeks between the initial meeting of the EPIC study working group and the validation and availability of the final study documents). The experience of the stakeholders involved in the project, regarding the specificities of EPIC as a community-based research project and the context of a global health crisis, will be explored in a knowledge management project led by the knowledge management department at Coalition PLUS.

### Mobilization, Capacity Building, and Coordination

Several key factors related to the structure of Coalition PLUS and its mission may explain the reach and the anticipated breadth of the EPIC program data. The established geographic, thematic, and linguistic networks were advantageous to efficiently channel and coordinate information from the field. Through 2 programs (*convention programs*) that aim to structure and reinforce the local capacities of 5 geographical networks in terms of services, research, and advocacy, and cofunded by the Agence Française de Développement (AFD), local coordinators, and CBRL team members, had a key role in centralizing and communicating information regarding the EPIC program. The CBRL team members based in the 5 geographical regions were able to closely follow the local implementation with the participating organizations. The CBRL also provided support for member organizations that are not part of these 5 geographical networks, including the research network, RIGHT PLUS, and the (linguistic) Lusophone network.

Although most of the organizations have previously participated in research studies, or have had a coordinating role, for some organizations, EPIC was a first-time experience having an active and lead role in the implementation of a research study. This is the case for the association Bésyp in Benin, which collected quantitative data from >200 transgender people, >600 MSM, and >50 CHWs through EPIC surveys. However, it is important to note that most of the participating organizations had extensive experience in conducting research and were instrumental in sharing their experiences and good practices within their regional networks. Such exchanges among the partner and member organizations are fundamental to knowledge sharing and capacity building within and across the Coalition PLUS networks. As the coordinating organization of the Lusophone network, Grupo de Ativistas em Tratamentos in Portugal, had a lead role in training and providing technical support for the organizations and overall coordination of the EPIC studies within the network. Importantly, the EPIC program represented not only a first experience with community-based research for many of the Lusophone network organizations but also an opportunity to collectively work on a research project, and in turn reinforce the structuration of the new network (created in 2019) and collaboration with Coalition PLUS. The ALCS in Morocco, one of the founding members of Coalition PLUS and coordinating organization of the MENA geographical network, was the first to conduct an EPIC study in the region. The ALCS was therefore able to share first-hand experience regarding study implementation during the webinar on qualitative methods with the other partners who were still in the preparatory phase of the study.

A project to capitalize on the EPIC program is currently underway to study and document the achievements and results obtained, lessons learned and good practices, the specificity of this research project compared with an academic research project, and to identify the key factors that contributed to the success of the project at the level of the association, at the level of the regional, thematic, or linguistic networks as well as within Coalition PLUS.

### Innovation but Not Without a Cost

For the CBRL, this was the first experience in developing a community-based study within the context of a pandemic as well as developing a general study protocol that was made available to the members and partners of Coalition PLUS. As described elsewhere [34], the EPIC program was launched without a clear idea of how many organizations would implement the study or the human resources and financing needed to assure the technical support and follow-up generated from the high demand. Although the flexibility of the EPIC’s methodology likely explains, in part, its high uptake within the Coalition PLUS network, it also adds complexity to the overall study coordination and follow-up. The global coordination of the study necessitated the mobilization of all 15 members of the research team, in addition to the coordinating organizations of the Coalition PLUS networks. Depending on the level of experience of the participating organizations, customized support regarding training on good study practices, data collection and management, and analysis were provided. The technical support provided by the Coalition PLUS CBRL will continue as participating organizations prepare to disseminate the results. Other Coalition PLUS departments, such as advocacy, knowledge management, and services, will have greater roles as results are used to inform recommendations and better practices.

Another significant factor to be considered is the fact that the development of the study protocol and its implementation in 2020 was possible largely because of the Coalition PLUS’ or partner organizations’ own funds. The number of participating countries greatly expanded in 2021 thanks to a reallocation of funds to pandemic efforts within the context of the *convention-programs* financed by the AFD [34]. Although 4 attempts were made to obtain research grants, success was limited because of the innovative methodology and the fact that research funding bodies were reluctant to fund studies that had already been launched at the time of the grant application. Research funding bodies also insisted on a justification for the choice of participating countries, the study populations, and sample sizes. This community-based research study was therefore evaluated within the framework of traditional research studies and not within the context of an ongoing pandemic, which explained the necessity to implement a flexible methodology and certain choices regarding the study population and size. The balance between keeping the research focus on responding to local needs during the health crisis and limiting local implementers’ potential exposure to COVID-19 while assuring scientific rigor has been touched upon elsewhere [35]. In addition to cofunding from the AFD, funding from the French Agency for Research on AIDS and Viral Hepatitis (*ANRS-Maladies infectieuses émergentes*) was secured to cofinance the qualitative data collection, analysis, and scientific dissemination of the EPIC program in the MENA region. Finally, funding was also secured from the Robert Carr Fund for the organization of 3 workshops for the presentation and discussion of the EPIC results from the West African and MENA regions. Minor local financing or other support for the EPIC program was obtained by some participating organizations through national institutions or through the contribution of resources from universities.

### Strengths and Limitations

Impelled by the urgency to identify and document emerging needs among KP at the start of the COVID-19 health crisis, the EPIC research program was developed following alerts from Coalition PLUS member and partner organizations. It was a research program guided by the principles of community-based research at its start and will continue to be throughout the research process. In line with the community-based research approach, the study populations and themes covered in the questionnaire or interviews were determined by the partner organizations in accordance with local needs. Therefore, the major strength of this program is the relevance of the study results to the local context. In addition, data regarding the impact of the COVID-19 crisis among KP at the local, regional, and global levels are particularly important to provide visibility to populations that are rarely represented in general population surveys. Furthermore, the EPIC data will capture precious information regarding the innovations or solutions that were put into place to maintain services during the crisis. This information could help support the sustainability of these solutions outside the COVID-19 crisis. The Coalition PLUS geographical and thematic networks aided in the (regional) coordination and implementation of the study. The collection of quantitative and qualitative data will provide more robust data to explore the experiences of KP and CHWs. Finally, implementation of this study, particularly by organizations that had little or no prior experience leading the local implementation of a research study, provided an opportunity for capacity building in research among the Coalition PLUS member and partner organizations.

The development and implementation of the EPIC program during a global pandemic has created several limitations that should be considered. COVID-19–related restrictions resulted in delays for ethics approvals and complicated data collection for some partners, sometimes resulting in a data collection period that spanned several months. Information regarding the state of the COVID-19 pandemic (eg, the epidemiological situation and governmental measures in place) at the time of data collection will be considered in the analyses to minimize potential bias. As each participating organization was able to construct their questionnaire or questionnaires according to local needs, there may be some limitations regarding the themes that can be explored in the global analyses. Thematic, regional, and global analyses will be performed whenever possible. Finally, some analyses will be limited owing to the sample size. Although it may not be possible to perform advanced data analyses in such situations, descriptive analyses will be performed and nonetheless will contribute important contextual information to broader common themes [37]. Analysis may also be limited owing to the quality of the collected data, particularly for qualitative data collection as it was a first-time experience in qualitative methods for many participating organizations. Although the literature on the implementation of multicountry qualitative research studies by previously untrained local implementors is limited, previous work suggests that implication of the local agents at the start of the study and continued support through the research process (including analysis) will help bolster the quality of the results [38]. In situations where the quality of EPIC data is not sufficient for in-depth analysis, efforts will be made to provide detailed feedback to the participating organizations to identify better practices.

### Conclusions

The multicountry community-based EPIC program was developed by various stakeholders, including and with the continued engagement of communities at all stages of the research process. With the challenge of documenting the impact of the global health crisis among KP and the CHWs who work with these populations, a general protocol was developed with a built-in flexibility that allowed the adaptation of the study to meet local needs. The deployment of this community-based research program within the Coalition PLUS network created the opportunity to not only collect data on the COVID-19 crisis but also to develop or reinforce capacity-building initiatives among the participating organizations through trainings, experience sharing, and continued technical support.

The EPIC program will provide essential information regarding the impact of the health crisis on KP at higher risk for HIV who are still little or not represented, depending on the geographical area, in the scientific literature. These results will therefore enhance community responses by capitalizing on new adaptations and innovations to HIV and viral hepatitis prevention and care services that may be pertinent beyond the current health crisis.

Finally, these data will also be essential to carry out national and international advocacy to ensure that KP are not forgotten in public responses to the current and future health crises. Thanks to the community expertise acquired in the field of HIV and hepatitis, it is anticipated that these data will show that the community-based response can be complementary to the public health response in a pandemic context and can continue to provide pertinent solutions for KP.

## Abbreviations

AFD: Agence Française de Développement
ALCS: Association de lutte contre le Sida
CBO: community-based organization
CBRL: Community-based Research Laboratory
CHW: community health worker
EPIC: *Enquêtes Pour évaluer l’Impact de la crise sanitaire covid en milieu Communautaire*
HCV: hepatitis C virus
KP: key populations
MENA: Middle East and North Africa
MSM: men who have sex with men
SW: sex worker

## Appendix

Multimedia Appendix 1 Ethics approval obtained by each participating organization.
