# Supplementary material for: Impact of the COVID-19 Health Crisis on Key Populations at Higher Risk for, or Living With, HIV or Hepatitis C Virus and People Working With These Populations: Multicountry Community-Based Research Study Protocol (EPIC Program)
Source: JMIR Res Protoc. 2023 Dec 14;12:e45204. doi: 10.2196/45204 (PMC10755648; doi:10.2196/45204)
Supplement: Multimedia Appendix 1 [file resprot_v12i1e45204_app1.docx]

**Multimedia Appendix 1**

Ethics approval obtained by each participating organization:

Algeria: University of Oran , 107DAPGR/CED/2021, CHU Annaba, 242/1630;

Angola: Comité de Ética Ministério da Saúde , N°33/2020;

Argentina: COMITÉ DE ÉTICA EN INVESTIGACIÓN, 2455;

Benin: Comité National d'Ethique pour la Recherche en Santé (CNERS), 31 27/08/2021;

Bolivia: Comité de ética y bioética de la facultad de medicina. Universidad Mayor de San Simon., Acta del 08 de abril de 2021;

Brazil: Comitê de Ética em Pesquisa em Seres Humanos da Santa Casa de São Paulo, 4.449.983; Burkina Faso: Comité d'éthique pour la recherche en santé, CohMSM PrEP: N°2017-7-105 & Réf A14-2021/CEIRES;

Burundi: Comité national d'éthique pour la protection des êtres humains sujets de la recherche biomédicale et comportementale, CNE/21/2021;

Cape Verde: Comissão Nacional de Protecção de Dados, Ref N°791CNPD/2020;

Chile: UNIVERSIDAD DE CHIL E - FACULTAD DE MEDICINA, COMITÉ DE ÉTICA DE INVESTIGACIÓN EN SERES HUMANOS, Proyecto: N° 204-2020 Archivo acta: N° 143;

Colombia: FEDESALUD: COMITE DE ETICA, No. 1 de 2021 & Comité Institucional de Etica en Investigacion, Universidad El Bosque, No. 008--2021;

East-Timor: Comité de Ética/Ténicnico do Instituto Nacional de Saúde (INS), No.Ref.71/MS-INS/DE/I/2021;

France: Comité d'Evaluation Ethique de l'Inserm, Institutional Review Board de l'Inserm (IRB00003888, IORG0003254, FWA00005831), Avis n°21-791;

Guatemala: Comité institucional de etica (CIE), Acta No.98 CIEREV106/2021;

Guinea-Bissau: Comíté Nocionol de Etico em Soúde (CNES), No Ref 098/CNES/INASA/2020;

Ivory Coast: Comité national d'éthique et de la recherche, CohMSM PrEP: N°088/MSHP/CNER-kp; Lebanon: University of Beiruth, USJ-2021-129;

Malaysia: Medical Research Ethics Committee, University Malaya Medical Center, 202121-9782;

Mali: Comité d'Ethique de la FMOS/FAPH, n°2020/130/CE/FMOS/FAPH & CohMSM PrEP: N°2017/113/CE/FMPOS;

Mauritania: Ministère de la santé/Comité d'éthique provisoire de la santé, 000580;

Mauritius: Republic of Mauritius
